# Perioperative Antithrombotic Management During Gastroenterological Surgery in Patients With Thromboembolic Risks: Current Status and Future Prospects

**DOI:** 10.7759/cureus.23471

**Published:** 2022-03-24

**Authors:** Takahisa Fujikawa

**Affiliations:** 1 Surgery, Kokura Memorial Hospital, Kitakyushu, JPN

**Keywords:** thromboembolic complication, bleeding complications, perioperative management, antithrombotic therapy, gastroenterological surgery

## Abstract

Antiplatelet medicines and anticoagulants are two types of antithrombotic pharmaceuticals, with anticoagulants including warfarin and direct oral anticoagulants (DOACs). During the perioperative phase, patients receiving antithrombotic therapy must balance two risks: bleeding and thromboembolism. To date, there are no defined recommendations for antithrombotic drug management in gastroenterological (GE) surgery, and the management strategy varies greatly between hospitals. The perioperative treatment of antithrombotic medications should be centralized according to the mechanism of each drug, and a suitable management strategy should be established. The proposed perioperative management for patients undergoing antithrombotic therapy is as follows: (1) in the case of antiplatelet medication, aspirin monotherapy is continued; (2) for patients on warfarin, it is substituted by DOAC bridging (preferred) or heparin bridging; and (3) in the case of DOACs, the short-term withdrawal of DOACs (typically 1-2 days) without heparin bridging is indicated. In the current review, the current state and future prospects of perioperative antithrombotic medication treatment during gastroenterological surgery are discussed.

## Introduction and background

Cerebrovascular disease and heart disease, along with cancer, are the major causes of death worldwide. With the emergence of an aging society in recent years, individuals with cardiovascular and/or cerebrovascular disorders are increasingly likely to require noncardiac surgery. In the majority of them, antithrombotic treatment (ATT) is used to prevent thromboembolism.

**Figure 1:**
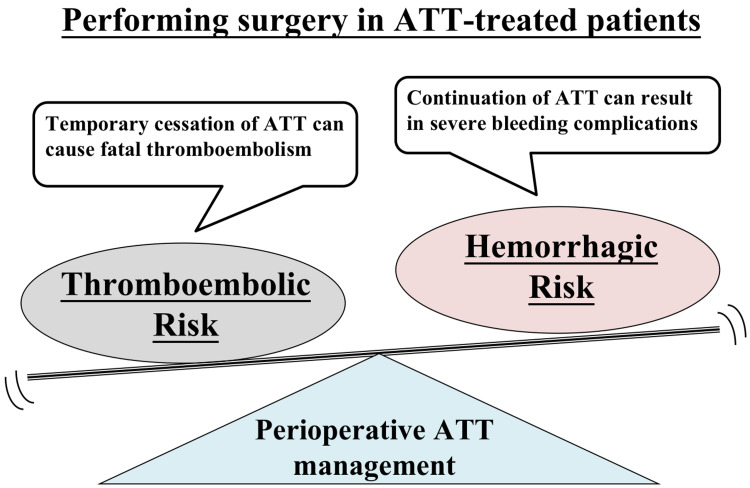
Antithrombotic management of patients receiving antithrombotic therapy (ATT) Perioperative continuation of ATT in ATT-treated individuals can result in serious bleeding problems; brief withdrawal of ATT can result in lethal thromboembolism. A compromise must be struck between minimizing the risks of thromboembolism and bleeding. Abbreviations: ATT, antithrombotic therapy.

Anticoagulation therapy (ACT) and antiplatelet therapy (APT) are two types of ATT. ACT involves the use of direct oral anticoagulants (DOACs) (synonymous with non-vitamin K-antagonist oral anticoagulants (NOACs)) and warfarin. Even in less invasive surgeries such as odontologic procedures, discontinuing antithrombotic medicines during the perioperative phase increases the risk of thromboembolism in patients receiving ATT, while continuing antithrombotic agents preoperatively increases the risk of bleeding (Figure 1) [1,2]. It is very vital to strike a balance between preventing thromboembolism and bleeding in gastroenterological (GE) cancer surgery, which is considered a highly invasive surgical technique.

According to the most recently updated guidelines for antithrombotic care during noncardiac surgery, preventing life-threatening thromboembolic events is more important than preventing bleeding problems [3-5]. However, there are no guidelines or evidence for ATT in GE cancer surgery. Our hospital is a high-volume tertiary referral center for patients with digestive cancer who require ATT, with ATT being used in 40%-50% of all surgical patients [2]. As a result, we developed a perioperative antithrombotic management protocol that we have been using since 2009, which includes the continuation of preoperative aspirin monotherapy for thromboembolic-risk patients [6,7]. An institutional guideline for GE surgery in patients receiving ATT was developed and is currently in use based on this procedure. The institutional guidelines have been modified as "Kokura Protocol 2019," with an appendix on DOACs [8].

The recommended perioperative antithrombotic care for patients who received ATT is outlined in this article, with special emphasis on GE surgery and laparoscopic or robotic procedures.

## Review

Antithrombotic management during elective GE surgery

**Figure 2:**
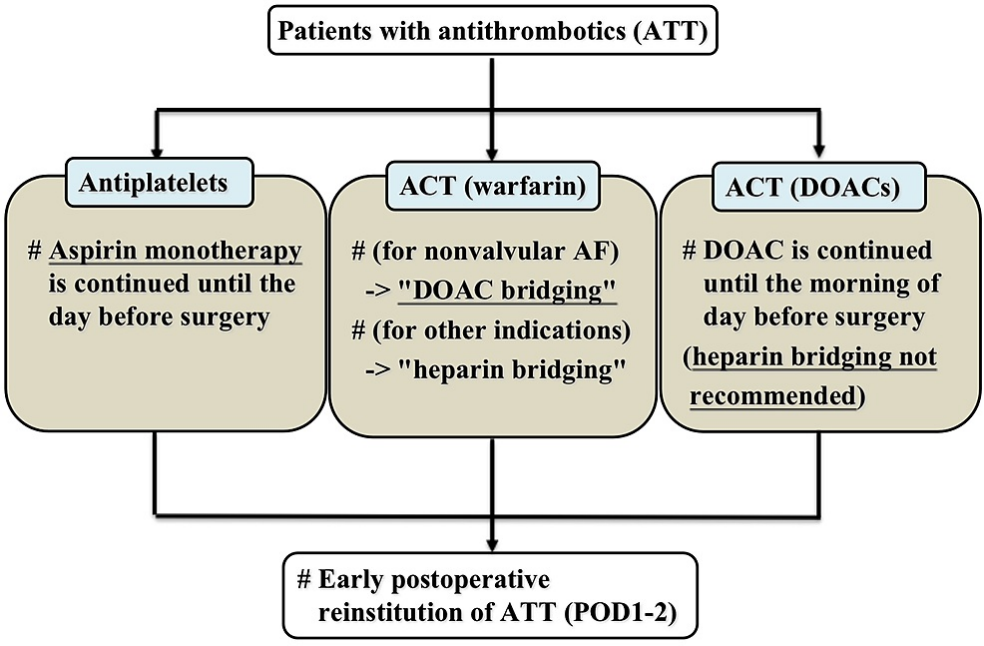
"Kokura Protocol 2019" The "Kokura Protocol 2019" is a proposed perioperative therapeutic regimen for patients taking antithrombotic medication in the event of planned gastroenterological surgery [8]. Abbreviations: ACT, anticoagulation therapy; AF, atrial fibrillation/flutter; ATT, antithrombotic therapy; DOAC, direct oral anticoagulant; POD, postoperative day

In the perioperative period during elective GE surgery, it is necessary to perform perioperative management according to the mechanism of each antithrombotic agent (Figure 2). The perioperative antithrombotic management for antiplatelet, warfarin, and DOAC is discussed here.

Antiplatelet Management During GE Surgery

When using antiplatelet medicines, the "Kokura Protocol 2019 [8]" recommends continuing aspirin until the day before surgery and then restarting it shortly afterward (Figure 2, left section). Medications other than aspirin are discontinued for individuals using several antiplatelet drugs, and surgery with aspirin is contemplated. If the risk of thromboembolic complications is significant in patients who are taking medications other than aspirin, such as thienopyridines, change them to aspirin and continue aspirin preoperatively before returning to the original agent early after surgery ("aspirin bridging"). When two or more antithrombotics are being used, early reinstitution is done in phases while verifying that there is no postoperative bleeding. Withdrawal before surgery may be undertaken if the risk of thromboembolism is minimal, but it is preferable to continue on a single aspirin as much as possible [8].

In terms of perioperative APT management, recent guidelines from the American College of Cardiology/American Heart Association and the European Society of Cardiology recommend that patients with a high risk of thromboembolism continue to take aspirin monotherapy at least during the perioperative period [5,9]. The focus has switched from the danger of gastrointestinal bleeding to the risk of thrombosis associated with antithrombotic drug cessation in recently updated guidelines from the American Society for Gastrointestinal Endoscopy and the Japan Gastroenterological Endoscopy Society [3,4]. In the case of GE surgery, however, there is no specific standard or practice for APT treatment in patients undergoing major GE surgery.

Two retrospective cohort studies employing a large number of cases were reported on laparoscopic GE surgery [6] and open GE surgery [7] to provide evidence for the safety of preoperative aspirin treatment during GE operations. Preoperative aspirin monotherapy did not affect the occurrence of surgical blood loss or postoperative bleeding complications in various types of GE surgery, including pancreatic surgery [10], liver resection [11,12], laparoscopic cholecystectomy [13], and laparoscopic colorectal surgery [14], according to recently published studies. Furthermore, Fujikawa et al. found that stopping APT before surgery was the most important risk factor for perioperative thromboembolic events and that continuing aspirin before surgery lowered the risk of thromboembolism considerably [2].

In the clinical situation, however, when the risk of thrombosis is severe, certain institutions may be directed to replace APT with heparin perioperatively. Heparin's mechanism of action differs from that of other antiplatelet medications. Moreover, the use of heparin is now well recognized as a significant risk factor for postoperative bleeding and should be avoided if feasible [7,15]. As a result, heparin bridging should not be used during APT cessation.

Although using a single aspirin in the perioperative phase is not linked to an increased risk of major bleeding issues [12,14], using several APTs or a combination of ACT and APT in surgical patients is more difficult. In a meta-analysis of large studies, dual APT with clopidogrel and aspirin was connected to a higher risk of severe bleeding [16]. As a result, in the case of several antithrombotics, early reinstitution should be done in stages rather than all at once to ensure that no bleeding episodes occur. Aspirin has only been reported as a preferred alternative for the perioperative treatment of continuing "single" antiplatelet medication in multiple published articles, as discussed above, and there is limited evidence for other antiplatelet drugs such as clopidogrel or prasugrel to date. For the safe perioperative treatment of these novel antiplatelet medicines, more research employing large-scale trials with an acceptable design is needed.

Patients who have just had a drug-eluting coronary stent placed or who have recently experienced thromboembolism are at an exceedingly high risk of myocardial infarction or thromboembolism, especially in the first month, making antithrombotic medication management challenging. Especially in patients undergoing recent coronary stenting, in order to balance the prevention of thromboembolism and the reduction of bleeding risk, at least a one-month waiting period should be set after coronary stenting, and then, GE operations should be conducted under perioperative continuation of aspirin monotherapy.

Warfarin Management During GE Surgery

In cases of non-valvular atrial fibrillation (AF) or a history of thromboembolism, the "Kokura Protocol 2019 [8]" recommends that surgery be conducted under transient replacement to DOAC ("DOAC bridging") for patients using warfarin (Figure 2, center section). Warfarin is replaced with heparin before surgery for other diseases such as valvular diseases and status following a mechanical valve replacement or for individuals with significant renal failure ("heparin bridging"). If the risk of thrombosis is minimal, but the risk of bleeding is high, warfarin discontinuation without the use of a DOAC or heparin bridging may be an option. The procedure should, however, be preceded by a full discussion of the risk of thromboembolism during warfarin cessation [8]. 

Heparin bridging has long been seen in patients using warfarin during the perioperative phase, but emerging data reveals that it is an important predictor for postoperative bleeding [7,15]. As a result, heparin replacement should be avoided to the maximum extent feasible.

The findings of the perioperative care of 1,884 warfarin-received patients with AF who had different therapies and procedures ("the BRIDGE trial") [15] were published in a multicenter prospective cohort study in 2015. The frequency of postoperative problems was examined in this study between a group that only stopped taking warfarin five days before surgery and a group that used heparin bridging. The findings revealed that, while there was no significant difference in the rate of thromboembolic complications, the heparin bridging group had a significantly higher rate of postoperative bleeding episodes. As a result, heparin bridging is not advised for individuals who are on warfarin for AF. However, it is unknown if individuals undergoing invasive surgery, such as GE cancer surgery, may be treated without heparin bridging. In reality, the majority of the 1,884 cases in the BRIDGE trial were treatments with minimal invasiveness, such as endoscopic procedures, with only 10 GE operations. As a result, the findings of the BRIDGE trial should not be extended to major GE surgery, and strict heparin bridging or, alternatively, DOAC bridging should be considered during warfarin discontinuation.

In 2017, a supplementary edition of the guideline for ATT therapy for gastrointestinal endoscopy incorporated a description of DOACs, and in patients with nonvalvular AF or a history of thromboembolism, DOAC bridging is preferred during warfarin withdrawal [17]. DOAC bridging is presently the main choice for perioperative care of warfarin patients since DOACs are more effective and safer than warfarin. Heparin bridging is planned in the event of additional criteria (e.g., status after cardiac valve replacement or valvular diseases) or in patients with severe renal problems, since the use of DOAC is confined to non-valvular AF or history of thromboembolism.

DOAC Management During GE Surgery

The "Kokura Protocol 2019 [8]" states that in the event of administering DOACs (Figure 2, right section), DOACs should be taken until the day before surgery and then discontinued. The withdrawal time may be extended in individuals who have a poor renal function or who have additional risk factors for bleeding. The medication is resumed early after surgery. Heparin bridging is not always essential, although it may be considered in some circumstances, such as when the risk of thromboembolism is quite high [8].

DOACs have been licensed for use in patients with non-valvular AF or a history of thromboembolism, and they are currently available. In the clinical environment, there are four kinds of DOACs now available. Only dabigatran inhibits direct thrombin, while rivaroxaban, apixaban, and edoxaban block factor Xa. DOACs are now being utilized more often as safe alternatives to warfarin. Although hypersensitivity events such as eczema or bronchospasm have been documented with DOACs [18], they are "easy-to-use" medications with a wide range of safety. Only dabigatran can currently be neutralized by a neutralizing agent. Large-scale randomized controlled trials have found substantial evidence for all four DOACs, and the meta-analysis findings from all four studies have been published in Lancet [19]. DOACs have much better effectiveness (inhibiting thromboembolism) than warfarin, and their safety (inhibiting bleeding events) is comparable to warfarin.

Heparin bridging was originally recommended in gastrointestinal endoscopy guidelines [4], and heparin administration during noncardiac surgery is also recommended in the 2013 AF therapy guidelines [20]. DOACs, on the other hand, may have advantages such as a faster start and offset of action [19], and the requirement for the perioperative use of heparin bridging remains a matter of debate. As a result of the BRIDGE study or others [7,15], the major negative effects of heparin bridging (enhanced postoperative bleeding) were later identified, and guidelines were modified in both Europe and the United States; presently, perioperative heparin bridging during DOAC cessation is not recommended [21,22]. In 2017, the Japanese gastrointestinal endoscopic recommendations were amended, including an appendix on DOAC treatment, and heparin replacement during DOAC discontinuation is not suggested, even in the case of high-risk operations [17].

Furthermore, the PAUSE research [23] looked at the results of 3,007 adult patients with AF who had DOAC treatment and had elective surgery or procedure. The DOAC treatment was stopped 1-2 days before the operation or procedure and restarted 1-2 days thereafter. The risk of significant bleeding was 0.90%-1.85% 30 days after surgery or procedure, and the incidence of arterial thromboembolism was 0.16%-0.60%. Although the participants in this research experienced a relatively limited number of major GE operations, the findings revealed that a consistent perioperative DOAC treatment strategy without heparin replacement may be utilized safely for patients with AF who receive surgery. In the case of elective GE surgery, Fujikawa et al. recently found that DOAC treatment with the use of heparin was the most important risk factor for bleeding issues and that perioperative heparin bridging was not advised even for patients undergoing major GE surgery [24].

Antithrombotic management during emergency GE surgery

**Figure 3:**
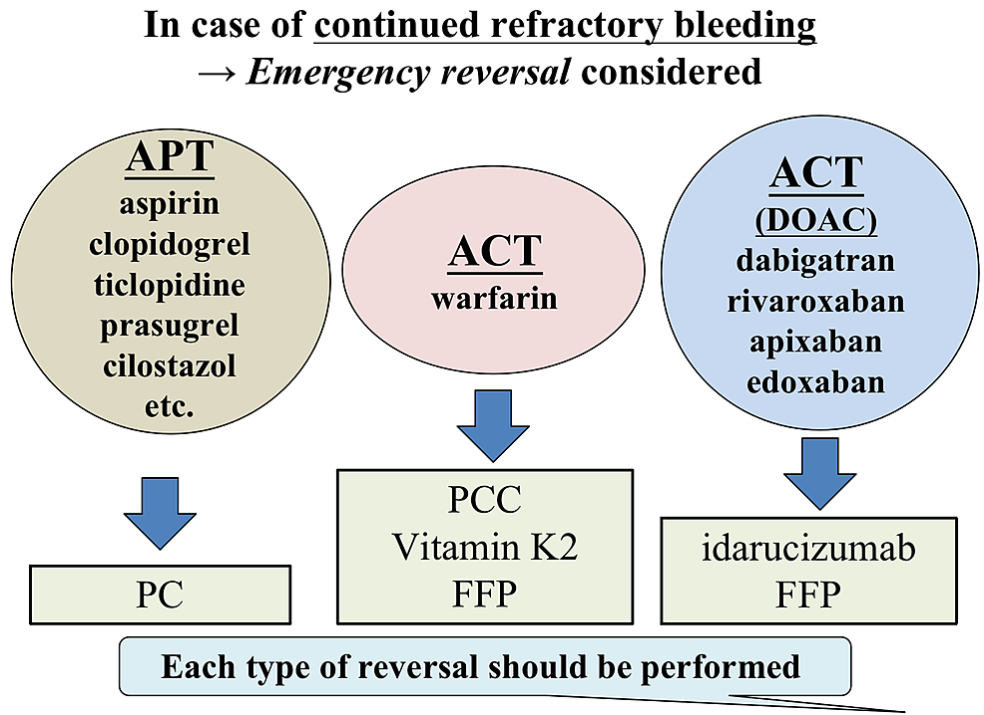
Antithrombotic therapy (ATT) reversal in an emergency Emergency reversal of each ATT type should be undertaken if refractory bleeding persists. Abbreviations: ACT, anticoagulation therapy; APT, antiplatelet therapy; DOAC, direct oral anticoagulant; FFP, fresh frozen plasma; PC, platelet concentrate; PCC, prothrombin complex concentrate

Because of recent advancements in endoscopic hemostasis procedures and equipment, the number of emergency operations for bleeding diseases has dropped. However, despite an increased risk of thrombosis, immediate reversal of the action of ATT may be necessary when ATT-burdened patients with refractory and problematic bleeding require emergency surgery. In this instance, it is necessary to reverse the antithrombotic effect according to each drug's mechanism (Figure 3). Platelet transfusion should be considered in the event of APT. When it comes to ACT, it is important to think about warfarin and DOAC independently. If the PT-INR is extremely high when taking warfarin, the first line of treatment is prothrombin complex concentrate (PCC) (Kcentra^TM^, CSL Behring GmbH, Marburg, Germany) plus vitamin K2 [25]. If PCC is not available, fresh frozen plasma is utilized instead of PCC. Only a neutralizer against the action of dabigatran (idarucizumab) is presently employed in the case of DOACs, and it can only be used for bleeding episodes during dabigatran therapy [26]. There is currently no neutralizer for the Xa factor inhibitor, and fresh frozen plasma reversal is being studied.

Meanwhile, the policy is not to perform emergency reversal in circumstances where intraoperative bleeding can be surgically managed in cases of urgent surgery other than surgery for intractable bleeding. Recent trends suggest that thromboembolism prevention should be prioritized, particularly in the case of emergency surgery [3,17]. Using proper energy devices to guarantee hemostasis, even in patients who received ATT, most emergency procedures may be completed without an increase in surgical blood loss. However, in the event of several antiplatelet medicines or warfarin with a high PT-INR, appropriate treatment, including emergency reversal, is required.

Laparoscopic/robotic surgery and antithrombotic therapy

Many GE and general surgeries are now done laparoscopically or with robotic assistance. Several studies have found that laparoscopic and robotic digestive surgery offer advantages such as faster recovery of digestive function, less body wall damage, less postoperative discomfort, fewer postoperative problems, and faster return to everyday life [27,28]. During laparoscopic and robotic surgery, reducing intraoperative blood loss and maintaining a dry operative area are crucial. Thanks to developments in numerous procedures and new surgical instruments, we are able to undertake a number of advanced laparoscopic GE surgeries. However, the best way to handle patients who get ATT during laparoscopic and robotic digestive surgery is still up for debate.

The risk of thromboembolic or bleeding complications was not substantially higher in patients with continued APT or heparin bridging than in patients with no ATT or interrupted APT, according to a systematic review of the results of laparoscopic GE surgery in patients undergoing ATT [29]. Another large-scale retrospective cohort analysis for various types of laparoscopic surgery showed that there was no significant difference in bleeding episodes between patients on continued APT and those who were not on APT continuation [6]. A retrospective cohort analysis of 258 liver resection patients found that, regardless of the size of the resection, laparoscopic liver resection is linked with lower surgical blood loss in both the ATT and non-ATT groups [11]. One case-control study utilizing propensity score matching analysis found that continuing aspirin during laparoscopic colorectal cancer resection had no effect on bleeding problems [14]. Although research into various forms of laparoscopic and robotic surgery is still underway, patients receiving ATT can safely undergo minimally invasive surgery, including laparoscopic and robotic surgery.

Recommendations for future studies

Although there are few reliable studies on the management of patients who received ATT during GE surgery, one multicenter, prospective, cohort study is currently underway (UMIN000038280, "Study on the safety and feasibility of gastroenterological surgery in patients undergoing antithrombotic therapy (GSATT study)") [30]. Well-designed analyses like this one will help clarify the safety and practicality of ATT management during GE surgery in the future.

## Conclusions

There are currently just a few credible studies on the treatment of patients who received ATT undergoing GE surgery, and management strategies differ substantially between facilities. The perioperative management of antithrombotic drugs should be centralized and tailored to each drug's mechanism. For patients undergoing ATT, the proposed perioperative antithrombotic management is as follows: (1) in the case of antiplatelet medication, aspirin monotherapy is continued; (2) for patients on warfarin, DOAC bridging (preferred) or heparin bridging is substituted; and (3) in the case of DOACs, the short-term withdrawal of DOACs without heparin bridging is taken into account. A defined approach or set of recommendations should be developed in the future based on trustworthy research with proper design.
